# Feasibility of an Application-Based Outpatient Rehabilitation Program for Stroke Survivors: Acceptability and Preliminary Results for Patient-Reported Outcomes

**DOI:** 10.3390/bioengineering11020135

**Published:** 2024-01-29

**Authors:** Annina Bindschedler, Carina Ziller, Eve-Yaël Gerber, Frank Behrendt, Björn Crüts, Katrin Parmar, Hans Ulrich Gerth, Szabina Gäumann, Wiebke Dierkes, Corina Schuster-Amft, Leo H. Bonati

**Affiliations:** 1 Strokecoach GmbH, 50670 Cologne, Germany; 2 Research Department, Reha Rheinfelden, 4310 Rheinfelden, Switzerland; 3 Faculty of Psychology, University of Basel, 4055 Basel, Switzerland; 4 School of Engineering and Computer Science, Bern University of Applied Sciences, 2501 Biel, Switzerland; 5 Department of Neurology, University Hospital Basel, 4031 Basel, Switzerland; 6 Department of Medicine, University Hospital Münster, 48149 Münster, Germany; 7 Department of Sport, Exercise and Health, University of Basel, 4052 Basel, Switzerland; 8 Department of Clinical Research, University of Basel, 4031 Basel, Switzerland

**Keywords:** stroke, rehabilitation, telerehabilitation, blended care, digital health, application-based training

## Abstract

Background: The majority of stroke survivors experience long-term impairments. Regular physical activity and other lifestyle modifications play an important role in rehabilitation. Outpatient rehabilitation using telemedicine might be suitable to improve functional ability and long-term secondary prevention. The Strokecoach Intervention Program (SIP, Strokecoach GmbH, Cologne, Germany) comprises training, coaching and monitoring with the aim of improving or at least maintaining functional independence and preventing further stroke through more targeted physical activity. The SIP is provided as blended care, which refers to the integrated and coordinated delivery of healthcare services that combines traditional in-person interactions with technology-mediated interventions, optimizing the use of both face-to-face and virtual modalities to enhance patient outcomes. Objective: The aim of this study was to evaluate the acceptance of the SIP by the participants and its practical application, as well as to obtain initial indications of effects of the SIP on the basis of patient-related outcome measures, blood pressure measurements and recording of physical activity in parallel with the intervention. Methods: Data from individuals with stroke participating in the SIP were analyzed retrospectively. Within the SIP, participants received an application-based training program, were instructed to measure their blood pressure daily and to wear an activity tracker (pedometer). During the intervention period of either 6 or 12 weeks, the participants were supported and motivated by a personal coach via a messenger application. The primary outcomes of the analysis were recruitment, acceptance of and satisfaction with the SIP. Secondary outcomes included functional measures, mobility and health-related quality of life. Results: A total of 122 individuals with stroke could be recruited for the SIP. A total of 96 out of 122 were able to start the program (54% female, mean age 54.8 (SD = 13.1), 6.1 (SD = 6.6) years after stroke onset) and 88 completed the SIP. Participants wore the activity tracker on 66% and tracked their blood pressure on 72% of their intervention days. A further analyzed subgroup of 38 participants showed small improvements in patient-reported outcomes such as health-related quality of life (SF-36) with an increase of 12 points in the subdomain mental health, vitality (12.6) and physical functioning (9.1). However, no statistically significant improvements were found in other performance-based measures (Timed Up and Go test, gait speed). Conclusions: This study showed that a blended therapy approach for stroke survivors with mild to moderate impairments in the chronic phase is feasible and was highly accepted by participants, who benefitted from the additional coaching.

## 1. Background

The incidence of stroke and the resulting burden on the health system have been increasing worldwide in recent decades [1,2]. Half of all stroke survivors do not fully recover and remain chronically disabled [3]. This affects their daily living, which can be seen in a low quality of life [4,5]. The areas of quality of life that are most affected are physical and mental well-being. [4]. Adapting to the new physical and mental limitations after a stroke can be overwhelming and therefore influences a patient’s well-being [5]. Schindel et al. showed that stroke survivors often have a lower quality of life than age-matched healthy individuals and that it remains low over time [5].

Most strokes are caused by modifiable risk factors [6], of which hypertension and obesity are the most important [7,8]. More than half of strokes worldwide are associated with high blood pressure [9]. Hypertension is not only an important risk factor for a first stroke, but also has a major influence on the occurrence of a second stroke [10]. Lowering blood pressure reduces the risk of stroke by almost one-third [11,12]. In addition to drug treatment, patients with hypertension can benefit from lifestyle changes. Poor diet and low physical activity are closely linked to high blood pressure and are therefore risk factors for a stroke [7]. People, who have moderate to high physical activity have a lower risk of having a stroke and associated mortality than people engaging in low physical activity [13,14]. This effect was observed across all age groups and genders [14]. Consistent engagement in regular physical activity not only serves as a preventative measure against the likelihood of experiencing a stroke but also exerts a profound influence on the severity of such cerebrovascular events. It has been demonstrated that individuals who partake in at least moderate exercise prior to a stroke tend to undergo strokes of reduced intensity compared to their inactive counterparts [15]. This underscores the pivotal role of physical activity in not only preventing the onset of a primary stroke but also in influencing its severity. The significance of physical activity extends beyond primary stroke prevention to encompass the mitigation of the risk associated with secondary strokes, while concurrently playing a central role in the overall rehabilitation process. Notably, a sedentary lifestyle exacerbates pre-existing disabilities [16], emphasizing the critical need to address inactivity in patients recovering from strokes. To achieve optimal rehabilitation outcomes, a tailored approach involving diverse exercises is indispensable. Such exercises should be carefully designed to target the specific areas affected by the stroke and effectively exploit neuromotor functions [17].

Furthermore, engagement in physical activity induces psychosocial enhancements by diminishing stress levels and exerting a positive impact on the mood of individuals, ultimately contributing to an improved quality of life for patients [16,17,18]. Implementing these requirements in a household setting can be challenging, so this is where the SIP comes into play.

For stroke survivors, it is recommended to engage in physical activities 20 to 60 min in three to seven sessions per week to reduce the risk for cardiovascular diseases [19]. Most individuals who have experienced a stroke typically only engage in limited and low-intensity daily physical activity, falling short of the recommended levels [20]. This is the case for both severely impaired and less impaired patients. Therefore, lifestyle interventions are needed to reduce the risk of a second stroke [20].

In recent years, the number of online applications for lifestyle changes has increased [21]. In particular, social distancing and restrictions due to the COVID-19 pandemic forced a rapid transition from conventional therapy in medical centers to online rehabilitation at home [22]. The advantages of digital interventions are that they can be offered to a large number of people at the same time, are tailored to patients’ needs and are cost-effective [23,24]. However, such applications must be easy to understand, as the use of smartphones can be a challenge for stroke survivors due to visual or cognitive deficits [25]. It has been shown that motor functions [26], as well as cognitive function in stroke, can improve throughout online therapy [25]. Results show that online therapy can have a similar effect on perceived performance, satisfaction, and difficulties in activities of daily living as conventional therapy [27]. Overall, online applications can support a seamless transition from inpatient to outpatient rehabilitation. Online-based therapy promises positive effects, but adherence to exercise programs is relatively low and the number of dropouts is high [23,27]. With app-based training in particular, it is important to have human interaction, such as messages or phone calls, in addition to online therapy [23]. Studies have shown that guidance from a therapist is one of the most important factors for patient adherence to therapy [28] as higher adherence is associated with a better outcome, especially in chronic patients [29]. Support when using the online application for the first time helps to minimize technical difficulties and barriers [28] and, additionally, the interaction with a therapist or a group has a positive effect on the perception of quality of life in stroke survivors [4,5]. Social support can lead to a more positive perception of physical condition, general well-being and overall perception of health [30]. It also strengthens self-efficacy, i.e., confidence in a certain behavior, which is highly associated with physical activity [31]. The concept of self-efficacy, which refers to an individual’s belief in the capability to execute and sustain specific behaviors necessary for achieving desired health outcomes, is central to the theory of supported health self-management [32]. It is based on the principle of empowering individuals to take an active role in managing their health and modifying behaviors conducive to well-being. Furthermore, the provision of instruction by a healthcare professional regarding the advantages and autonomous execution of a specific exercise can positively influence the motivation of individuals recovering from stroke to engage in the training [31]. Therefore, blended-care solutions that combine online and face-to-face therapy appear to be more effective than online therapy alone [23].

The SIP intervention was implemented with stroke survivors by the Strokecoach Company in March 2020 in in the greater region of North Rhine Westphalia (Germany). The aim of this study was to retrospectively evaluate the feasibility of application and acceptance to participating stroke survivors of the home-based Strokecoach Intervention Program, as well as to obtain initial indications of effects of the SIP on the basis of patient-related outcome measures, blood pressure measurements and recording of physical activity.

## 2. Methods

### 2.1. Study Design

The Strokecoach company (Strokecoach GmbH, Cologne, Germany) invited stroke survivors to take part. Pre–post measurements of patient-related and performance outcomes were performed. Each participant provided informed consent, granting permission for the utilization of their data in a subsequent and thorough analysis. The current study is a retrospective analysis of these data collected from March to August 2020. The ethics committee of the responsible medical association of North Rhine-Westphalia (Germany) stated that no approval was required for this retrospective study.

### 2.2. Participants and Setting of the SIP

The eligibility criteria for the inclusion of potential participants in the prior SIP were (1) diagnosis of prior stroke, (2) smartphone literacy, (3) aged 18 and older, (4) the self-reported ability to walk at least 10 m independently, (5) medically stable, and (6) living at home. Exclusion criteria were: (1) acute illness or other clinically significant diseases, (2) contraindications to physical training, or (3) inability to follow the study procedures. The eligibility criteria for program participation did not include factors such as the number of strokes, their duration, type, or site. The selection was made based on the information provided by the potential participants.

### 2.3. Participant Recruitment of the SIP

Potential participants were approached via different recruitment strategies: (1) a database screening was performed in a German hospital, and potentially interested persons were contacted. Other methods included (2) social media platforms (Facebook, Instagram), (3) online advertisements and (4) self-help groups, (5) direct contact through a German insurance company, (6) a private occupational therapy practice, and (7) through word of mouth. There were two recruitment phases. Participants enrolled in the first recruitment phase (58%) received a 12-week program, while those who started later only completed a 6-week program. This was due to internal and financial reasons.

### 2.4. The Strokecoach Intervention Program

**Figure 1:**
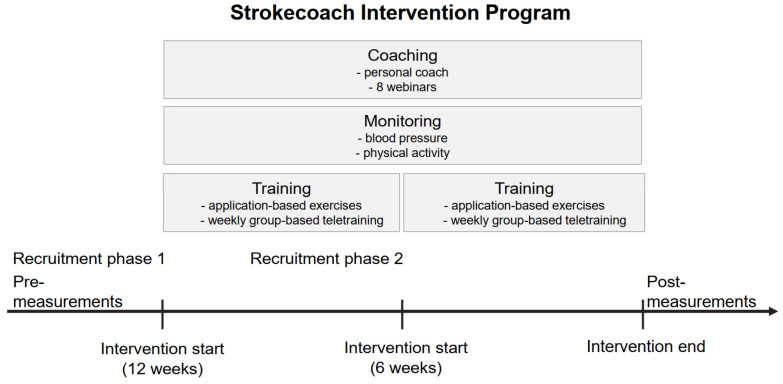
Strokecoach Intervention Program flow chart.

The Strokecoach Intervention Program has been developed to encourage stroke survivors to achieve a higher level of targeted exercise training at home, thereby improving their independence in daily life and reducing the risk of further strokes. The SIP (Figure 1, Table 1) combines training, coaching, and monitoring, lasted three months and was originally designed as a blended-care model, with participants training once a week in small groups, combined with an application-based exercise program at home. Due to the COVID-19 pandemic, which led to a complete lockdown and social restrictions in Germany for months, the SIP was changed to a telerehabilitation-only program that included a combination of home training and weekly online group sessions. The Strokecoach application [33] was developed by the Strokecoach company (Strokecoach GmbH, Cologne, Germany) and could be downloaded free of charge. All participants received an individual code for registration and if necessary, they were supported by their personal coach for technical assistance. The application itself was not modified or adapted during the intervention process. The intervention was conducted in the greater region of North Rhine-Westphalia (Germany).

Training part of the SIP: Training took place at home using the Strokecoach application. Upon launching the Strokecoach application on their smartphone or personal computer, participants were systematically led through their daily exercise regimen. The app contains a weekly overview, descriptions and videos of exercises, and short, motivating and educational inputs in text form. Videos of the exercises can be found online [34]. The individual training plan consisted of alternate exercises for the upper and lower extremities, as well as the trunk. Tailored exercise selection was based on the modified Rankin Scale [35] (mRS), which was self-rated by the participants based on questions about their individual extent of neurological impairment. The mRS focuses on impairment rather than diagnosis and thus provides a useful basis for exercise selection. For each exercise, the mRS level for which this exercise is suitable was specified. Daily and longer-term programs were automatically created on the mRS-classified exercises. If an exercise could not be performed, either an alternative exercise was selected or the execution of the exercise was adapted. Such an adjustment was made by the coach. However, the videos and descriptions also included variations in a movement form to take account of individual requirements. Intensity of the training was controlled by either the number of repetitions or the duration, and was automatically increased from week to week, also depending on the individual perception of the intensity. Once a week, supervised group-based tele-training took place to provide social support and help with the exercises. Following the intervention, inquiries were made to determine if there were any health issues during the intervention period that may have influenced participation.

Coaching: The main goal of coaching was to promote the motivation of the participants and to prevent them from feeling alone and aimless in their training at home. Each participant was assigned a personal coach. All of them were experienced occupational therapists. Their task was to inform, motivate, and provide support. For example, if questions arose regarding the execution of training exercises, the coaches could be contacted. On the other hand, each participant was contacted at least once a week by the coach. The support from the coaches was provided throughout the entire intervention period. In addition, participants were offered eight webinars in which they received advice on various health topics, such as balance, fall prevention, strength exercises, physical flexibility, sleep, and healthy eating (Table 1).

Monitoring included measurements of blood pressure and physical activity. All participants were thoroughly instructed regarding the measurement of their blood pressure using an upper arm blood pressure monitor (BU 540 Connect, Medisana, Neuss, Germany) and were asked to perform this three times in the morning in a resting position. Participants received a warning from their coach if the blood pressure was above normal for three consecutive days (e.g., systolic: >140 mm Hg). They were recommended to see their general practitioner to re-check or adjust medication. Categorization of the alerts was performed according to the three hypertension stages described in the literature [36,37]. No additional safety monitoring of the intervention was conducted. Blood pressure data were automatically transmitted and stored using the secure cloud solution offered by Philips’ HealthSuite Digital platform (Philips, Amsterdam, The Netherlands).

All participants had a wrist-worn activity tracker (Xiamoi Mi Band 3, Anhui Huami Information Technology Co., Ltd., Hefei, China) during the day, which counted the steps.

**Table 1:** Intervention description based on the TIDier checklist [38].

1. Brief name	- SIP, Strokecoach Intervention Program
2. Why Describe	- to combine different aspects of secondary prevention after stroke, including physical activity, education and monitoring of blood pressure
3. What Materials	- participants’ personal smartphone - Strokecoach application [33] - participants received a blood pressure monitor (BU 540 Connect, Medisana, Neuss, Germany) and an activity tracker (Xiamoi Mi Band 3, Anhui Huami Information Technology Co., Ltd., Anhui, China) via mail.
4. Procedures	(1) Training - on six days/week followed by a rest day - allocated training plan based on individual mRS level - exercises were chosen from a pool of 20 standard exercises (8 for the lower extremity, 9 for the upper extremity, and 3 for the trunk) as well as 82 variations in these exercises - online group exercise training once a week (45–60 min) - participants were advised to maximize their physical activity by enhancing steps per day (2) Coaching - communication with coach via messenger or e-mail (participants got at least one personal message per week) - eight online educational webinars (20 min): balance, fall prevention, strength exercises, physical flexibility, sleep, healthy eating, questions and answers, study results) (3) Monitoring over the intervention period - physical activity - blood pressure
5. Who provided	- occupational therapists experienced in treating people with neurological dysfunction - they received intensive training regarding the SIP
6. How	- intervention was delivered remotely via a smartphone application - digital group-based training once a week
7. Where	- participants trained in their personal home
8. When and how much	- training plan for a planned intervention period of six weeks on 6 days per week followed by a rest day - one training day consisted of approximately 10 exercises - online group exercise training took place once a week (45–60 min) - participants from the first recruitment phase were delivered two six-week exercise programs, whereas participants from the second recruitment phase received only one six-week program - training intensity was controlled either by the number of repetitions or by their duration
9. Tailoring	- the training plan was allocated according to the mRS level, and, if necessary, the exercises were tailored - Repetitions or duration were increased weekly unless participants indicated that the intensity should remain as it is
10. Modifications	- the intervention was not modified
11. How well: planned	- adherence to monitoring of physical activity and blood pressure was assessed (please see outcomes), exercise adherence was not tracked - participants were motivated with instructions via messenger to be physically active and to perform their exercises as instructed
12. Actual:	- if the participants did not exercise on one day, they could catch up on the missed exercise program the next day, which extended the intervention period - coaching evaluation forms were sent to sample of 54 participants

Legend: SIP = Strokecoach Intervention Program.

### 2.5. Outcome Measures

Sociodemographic and stroke-specific factors were collected via an online questionnaire and included sex, age, mRS level, type of stroke, time since stroke, recurrent stroke, affected side, affected areas, number and type of medication, and comorbidities. Additionally, the participants rated their paretic arm function, arm strength and arm pain on a 10-point rating scale. Possible answers ranged from ‘no function at all’, ‘no strength at all’ and ‘no pain at all’ to ‘I can do anything I want’, ‘full strength’ and ‘the worst pain I can imagine’.

For the retrospective analyses, primary outcomes were feasibility and acceptability endpoints comprising study procedures and patients’ acceptance of the SIP. Table 2 provides an overview of specified objectives, endpoints, and outcome measures.

Outcomes included the potential effects of the SIP on quality of life and self-performed tests. Online questionnaires (google forms) were sent to the participants at the start (pre) and end of the intervention (post). For the performance measures, instruction videos were sent.

The following outcome measures were used:

▪ Health-related quality of life was determined using the German version of the 36-item Short Form Survey (SF-36). The SF-36 consists of eight subscales: physical functioning, role limitations due to physical health, role limitations due to emotional problems, energy/fatigue, mental health, social functioning, pain, and general health. Each consists of different questions and the score ranges from 0 to 100, with higher values representing better health-related quality of life [39].
▪ The Timed Up and Go Test (TUG) is a well-known mobility test and is often used in stroke rehabilitation [40,42]. Participants were instructed to get up from a chair, walk a distance of three meters, turn around, and sit down again. They were advised to ask another person to measure the time.
▪ The 4-Metre Walking test was used to measure habitual gait speed. Although assessments of walking speed are common, different protocols are used [43]. In our study, patients were instructed to measure the time for walking a distance of 4 m. They started from a resting position and repeated the test four times, as previously described for people with COPD [41]. The mean was calculated.
▪ Muscle strength was evaluated by self-performed exercises based on the Medical Research Council Scale [44]. Participants rated their performance for shoulder flexion, elbow flexion, wrist extension, hip flexion, knee extension and foot dorsiflexion bilaterally on a 6-point rating scale.
▪ General health questions included the self-rating of capabilities walking, speaking, vision, memory, concentration, mood, sleep and current health on a 10-point visual analogue scale with verbalized endpoints (very bad to very good).
▪ For the evaluation of coaching aspects, online questionnaires were sent. Webinar attendance was evaluated dichotomously. The questions about their webinar experience were rated on a visual analogue scale (from “not at all useful” to “very useful”). Based on the Counselor Rating Form–Short Version (CRF-S) [45], the coach was rated on different items (friendly, experienced, authentic, sociable, competent, honest, sympathetic, reliable, empathic, helpful, expertly, trustworthy) with 0 = not at all to 5 = very much.

Blood pressure data, physical activity data and online surveys were collected on a secured web-based platform that was only accessible to the investigators.

### 2.6. Data Analysis and Statistics

Data analyses were performed retrospectively by members of the Research Department of the Reha Rheinfelden (Rheinfelden, Switzerland).

Participants’ characteristics, acceptability parameters and interview data were analyzed descriptively. As applicable, absolute and relative frequency, together with parameters of central tendency and distribution, were presented. For continuous data (blood pressure and physical activity), we divided the intervention time into weekly categories and calculated the mean values per week.

We analyzed data from a subgroup of patients for whom pre- and post-intervention measures were available from the TUG and SF-36 on the data from the TUG, SF-36 and the general health questions. Paired-samples *t*-tests were used for pre–post comparison and normality distribution was checked with the Shapiro–Wilk test. If the assumption of normality was violated, the Wilcoxon signed-rank test, a non-parametrical test, was used instead. The alpha level for statistical significance was set to 0.05. Statistical analysis was performed with R Statistical Software (RStudio, v2023.06.0+421, Posit Software, PBC) and JASP (v0.17.0, The Jasp Team, Amsterdam, The Netherlands).

## 3. Results

### 3.1. Feasiblity of the Stroke Intervention Program

**Figure 2:**
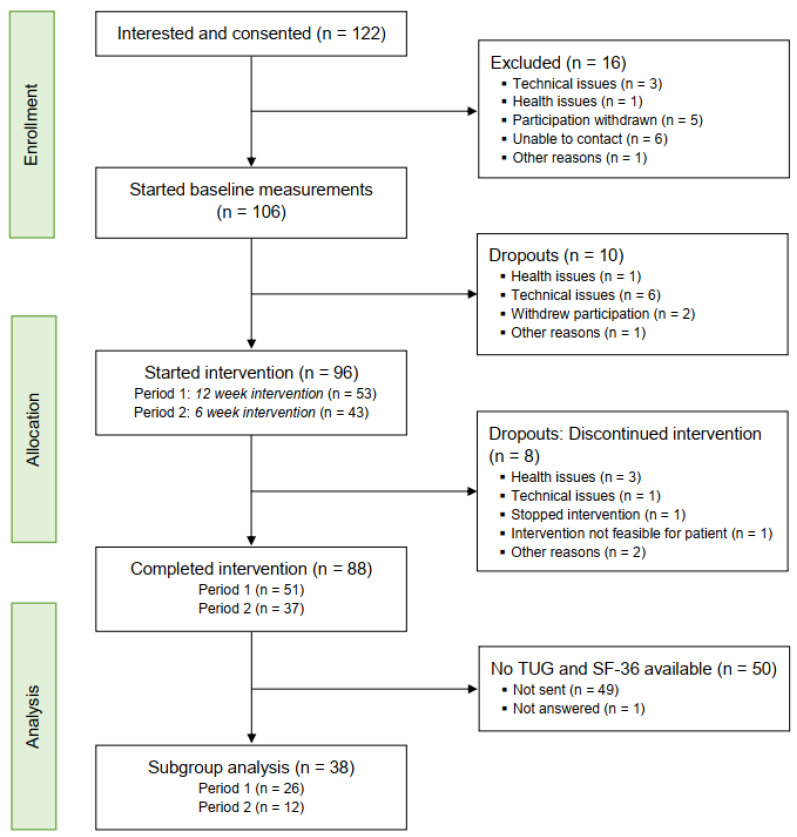
Participant flow diagram.

Figure 2 shows the flow of recruitment, enrolment and study completion. From the 122 initially screened individuals with stroke, who provided informed consent to participate, 16 were excluded or dropped out before baseline measurements started. Of the remaining 106 potential participants, 96 started the intervention and were considered to be enrolled in this study, resulting in a baseline measurement rate of 90.6%. Descriptive sociodemographic data of the participants are presented in Table 3. The majority of them had no or slight impairments (mRS level 1: 46.6%, mRS level 2: 27.1%). Some participants classified themselves as moderately disabled (mRS level 3: 16.7%) or moderate-severe disabled (mRS level 4: 9.1%).

Of the 96 enrolled stroke survivors, 32% of them were recruited via a private occupational practice, 24% via social media (Facebook, Instagram) or by the mailing list of the German stroke association Stiftung Schlaganfall-Hilfe (16%). Only a few were recruited by word-of-mouth recommendations (8%), via the Nuremberg health insurance (3%) and the Strokecoach website (1%). For 16% of the participants, it was not traceable. Of the 96 participants, 88 (91.7%) completed the intervention. This corresponds to an attrition rate of 8.3%. Three of them stopped the intervention due to health-related issues; one person was severely impaired and could not perform the exercises, although she categorized herself with mRS 3.

Subgroup characteristics are given in Table 4. Most participants rated themselves on the mRS as slightly affected (category 1: 28.9%, category 2: 60.5%, category 3: 7.9%, category 4: 2.6%). The average duration of the intervention was 92.6 days (SD: 25.1). This corresponds to 23 days more for recruitment phase 1 and 19 days more for phase 2. Most of the subgroup participants reported (multiple responses were possible) that the stroke had affected them in the areas of gait (79%), hand and arm function (71%) and concentration and/or memory (57.9%). Limitations persist concerning mood (39.5%), pain (36.8%), speech (34.2%), vision (31.6%) or others (15.8%). Around 85% of the subgroup reported comorbidities (multiple responses were possible). The most frequently mentioned comorbidities include diabetes, cerebrovascular diseases, rheumatologic diseases and musculoskeletal problems (18.5%, respectively). The self-reported number of daily medications was retrospectively categorized as no medication (13.2%), one to four (44.7%) and five or more different medications per day (42.1%). Most of them took antiplatelets (71.1%) and antihypertensive medication (55.3%). For a detailed description, please see the Supplementary Table S1.

### 3.2. Acceptability of Using the Strokecoach Intervention

For participants of the first recruitment phase (planned duration of 6 weeks), the actual average intervention time was 98.5 days (SD 18.7, range: 66–144 days). The patients from the second recruitment phase trained for longer than the initially planned 42 days. The duration was on average 61.6 days (SD = 5.9, range: 44–92 days).

Blood pressure measurements and physical activity monitoring were accepted by patients. Patients wore their activity tracker on 65.9% (SD = 28.2%) and assessed the blood pressure on 72.4% (SD = 45.3%) of the intervention days.

**Figure 3:**
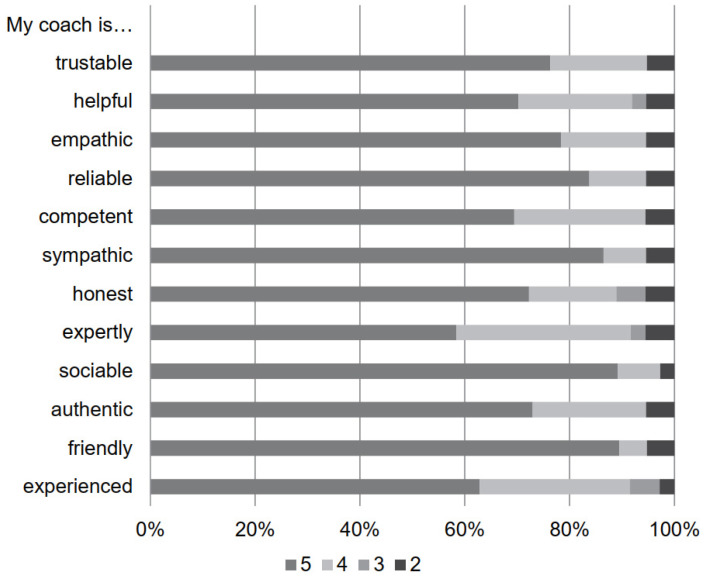
Evaluation regarding satisfaction with personal coach concerning various personal aspects. Legend: 6-point rating scale from 0 to 5, 0 representing not at all, 5 representing very much.

Webinar invitations for the SIP evaluation were sent to a sample of 54 patients. Most patients attended the webinars at least once (81%) and rated the duration of the webinars as just right (88%). The median of the overall satisfaction was 8 [IQR 2.75]. The median rating for the coach in all 11 categories was 5 (Figure 3).

### 3.3. Patient-Reported Outcome Measures and Performance-Based Measures

**Figure 4:**
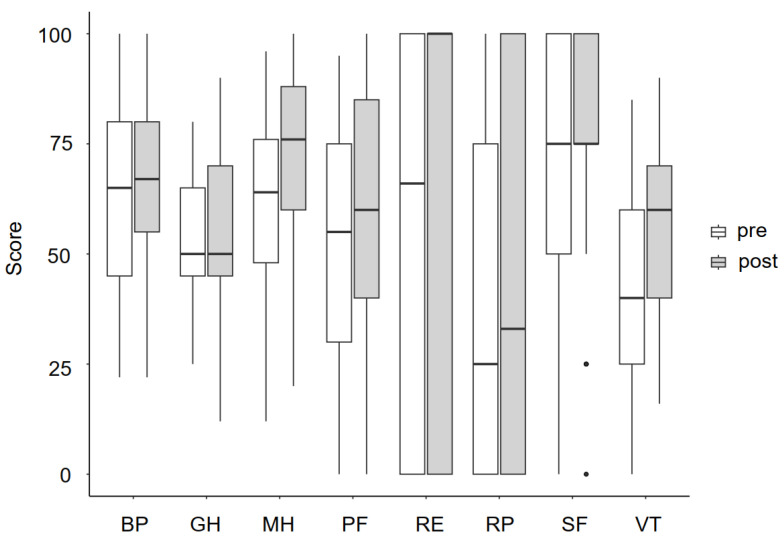
SF-36 scores pre- and post-intervention (*n* = 38). BP = bodily pain, GH = general health, MH = mental health, PF = physical functioning, RE = role limitations due to emotional health, RP = role limitations due to physical health, SF = social functioning, and VT = vitality.

Three dimensions of health-related quality of life significantly improved at the end of the intervention time relative to the baseline measures (Figure 4). The performed *t*-test showed that there is a significant difference in the dimensions of mental health and vitality between pre- and post-intervention conditions, with a mean improvement (location parameter) of 12 points and 12.6 points, respectively. Cohen’s d states that this is a large effect. Similarly, the Wilcoxon signed-rank test showed significant improvement in the dimension physical functioning. For exact values and statistics, please see Supplementary Table S2.

**Figure 5:**
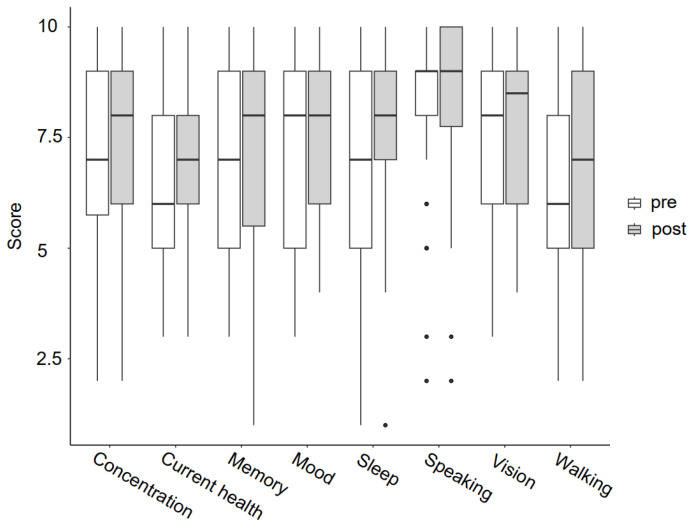
Answers to general health questions pre- and post-intervention (*n* = 38).

General health was determined by different questions. Medians on a 10-point rating scale for self-estimated walking ability, concentration and current health were slightly improved after the intervention (Figure 5). The Wilcoxon signed-rank test confirmed this trend. However, there was no change in speaking ability, mood, vision, sleep and memory function. For exact values and statistics, please see Supplementary Table S3.

Functional mobility and walking speed did not differ between pre and post-intervention measurements. Less impaired patients needed less time in the TUG and needed less time in the 4 m gait test. There were no clinical or statistically significant differences in the pre–post comparison. For exact values and statistics, please see Supplementary Table S4.

Statistical analysis (Wilcoxon signed-rank test) showed no difference in self-reported muscle strength measured with the MFC. As expected, the median in all MFC muscle tests of the non-affected side remained the same before and after the intervention (Md = 5). The same applies for the affected side for shoulder flexion, elbow flexion, wrist extension and foot lifting (Md = 5). Participants could slightly increase their self-reported strength in hip flexion and knee extension. However, this was not statistically significant.

### 3.4. Physiological Parameters and Physical Activity Patterns

**Figure 6:**
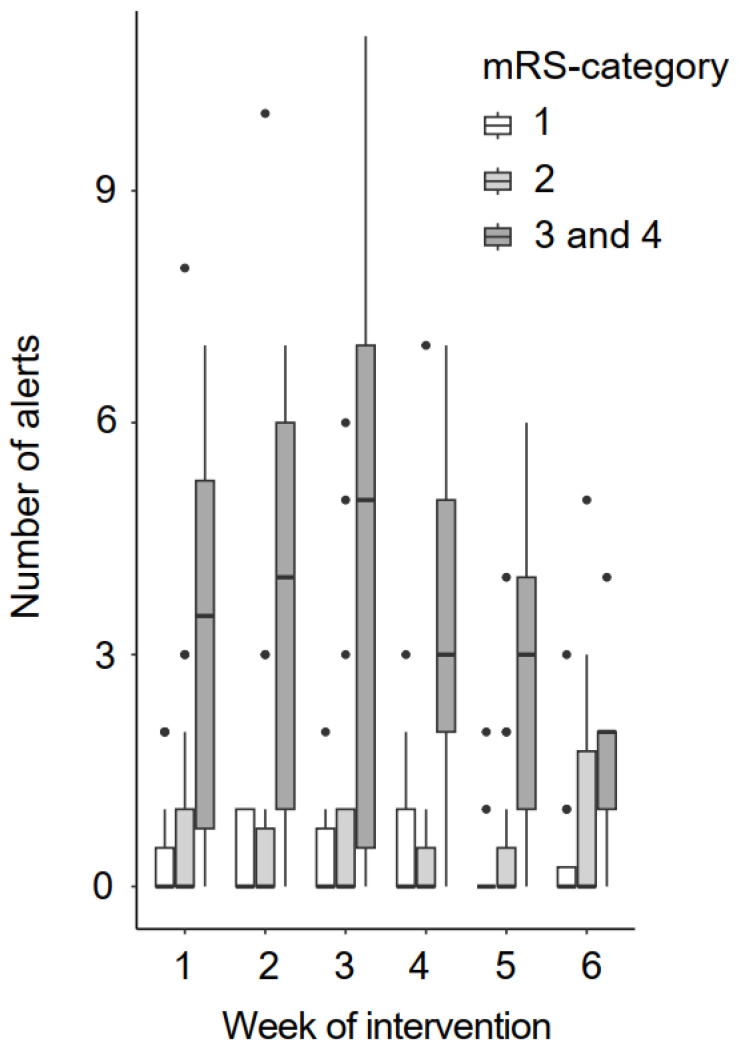
Number of blood pressure alerts per week. mRS = modified Rankin Scale.

The average number of blood pressure alerts per week for the participants depending on the mRS category is shown in Figure 6. They did not change over the intervention duration. On average, patients had an alert on 14.4% (SD = 17%) of their intervention days. In general, there were mainly alerts of hypertension stage 1. We could analyze the number of alerts for 6 weeks of the intervention period for 41 patients. In total, they had 1534 measuring days, with 225 alerts of hypertension stage 1, 14 alerts of hypertension stage 2 and only one alert of hypertension stage 3.

**Figure 7:**
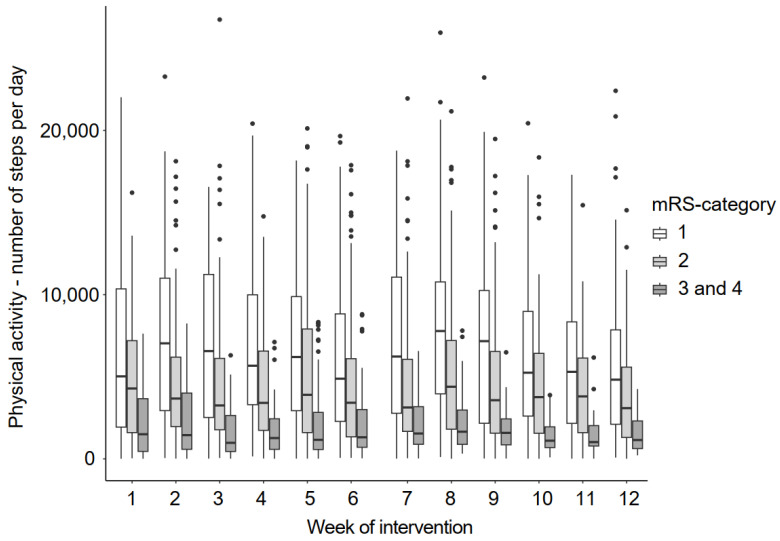
Physical activity during the intervention period. Legend: mRS = modified Rankin Scale.

In general, participants who were less affected walked more steps per day. There was no change in the number of steps per day over time (Figure 7).

## 4. Discussion

This study retrospectively evaluated data gathered during the implementation of a home-based exercise program for stroke survivors—the Strokecoach Intervention Program. The assessment of the SIP focused on practicality, patient-reported and performance-based outcome measures, as well as physiological and physical activity parameters.

The development of the SIP appeared to be an important step as there was a lack of high-quality, long-term prevention programs to reduce the burden of stroke [46]. Since the COVID-19 pandemic, an increasing number of studies have been conducted to evaluate tele-rehabilitation measures in stroke care [47,48,49]. However, only a few secondary prevention programs could show long-term effectiveness [50]. The SIP, designed as a blended-care program, followed a comprehensive strategy that included training, monitoring and coaching to address risk factors in secondary prevention. By monitoring both physical activity and blood pressure, significant risk factors in secondary prevention were addressed by the SIP [50,51].

A substantial number of the enrolled individuals with stroke completed the entire program, but a subset of the participants did not provide data from certain self-assessed patient-reported outcome measures. Nevertheless, participants monitored their daily physical activity for more than half of the intervention duration, which offered them immediate performance feedback, and the task of increasing their daily step count could have served as a motivating factor. The data revealed that a majority of the participants failed to reach the recommended daily step count of 6500 to 8500 for individuals with impairments or chronic diseases [52]. A minimum of 6000 steps has been associated with a diminished risk of encountering subsequent cardiovascular events [53]. The data on walking speed results show that less impaired participants with a lower mRS level were faster than more impaired participants. On average, the included participants exhibited deficits in walking speed and did not reach the threshold of 1.3 m/s, which is necessary for safely crossing a street [54]. A blended-care model, as it was used within the SIP, can promote physical activity [23]. Marsden et al. evaluated a pilot study involving a 12-week home-based exercise program for stroke survivors, incorporating telephone and email support, and observed a more substantial improvement in cardiopulmonary fitness compared to control groups [26]. Such models have also been successfully integrated into other therapeutic areas, such as musculoskeletal physiotherapy, and have shown promising results [55,56].

An important element of the SIP was the continuous monitoring of blood pressure. Home-based monitoring of blood pressure showed positive aspects, providing the advantage of detecting white-coat and masked hypertension [57], and promoting increased patient engagement in blood pressure measurement. In the present study, the feasibility and acceptance of blood pressure monitoring were demonstrated, with participants measuring their blood pressure on nearly 75% of the intervention days. Based on the data, it cannot be concluded that the SIP led to alterations in lifestyle or improvements in blood pressure values. However, it is known that after a stroke, blood pressure frequently remains uncontrolled. In a cross-sectional analysis of individuals after a stroke, over fifty percent exhibited uncontrolled hypertension three months after the incident [57]. Therefore, monitoring blood pressure is imperative.

Alongside monitoring, coaching was an essential component of the program, specifically in terms of motivation. This was evident in the high satisfaction of the participants with the coaches. In addition to the individual improvements in clinical parameters, participants identified motivational factors as the primary benefit derived from the SIP. This speaks to a potentially positive influence that the SIP may exert, considering the well-established importance of motivational factors in rehabilitation [58].

The SIP was designed with the objective of mitigating risk factors and enhancing strength and cardiorespiratory fitness of individuals after stroke, aspects relevant to independence and overall quality of life. However, in patient-reported outcome measures, the participants exhibited only slightly enhanced scores in various aspects relevant to daily life such as walking. Statistically significant improvements could be observed in the SF-36 subdomains of physical functioning, mental health, and vitality. The improvement in social and psychological outcomes indicated that, despite the primary focus on exercises, the SIP addressed not only physical aspects but also health-related quality of life and mental health. Vitality is a parameter of fatigue that was improved in this study. The mean difference in the vitality subdomain can be regarded as clinically relevant [59].

There was almost no improvement in performance-based measures, potentially due to the limitations of the selected assessments. For instance, the use of a stopwatch for patients to determine the time required during the gait tests certainly posed a challenge. While some participants sought assistance from another person, it was not obligatory. Further, the distance of four meters seems too short to measure gait speed and it has previously been described as possibly leading to misclassification [60]. Additional measures of physical activity, as suggested in the literature, including accelerometer-measured physical activity, sedentary time, and activity patterns, may offer more meaningful insights for discerning and assessing changes in cardiovascular risk [61]. For instance, utilizing an accelerometer offers a feasible choice for administering the TUG test in a home setting [62].

### 4.1. Strengths and Limitations

The SIP had some limitations. (1) The explorative approach of data collection using online forms resulted in a number of missing data. (2) During the recruitment process, individuals with stroke who were already active in self-help groups or therapy showed interest in participating. Therefore, volunteer bias must be considered and might explain the high level of acceptance and satisfaction with the SIP. The patients’ impairment level was rather low. (3) Therapy intensity and frequency were not tracked, so we cannot draw conclusions about actual exercise adherence. (4) Pre-measurement and post-measurement periods would be needed to address risk reduction. (5) The current study did not include an economic analysis. Therefore, nothing can be stated regarding a cost-saving effects of this program. However, it is known that telehealth interventions for care can save time and financial resources. (6) The duration of the intervention varied widely between the participants, from 44 to 141 days, due to recruitment in two different periods and the possibility of stopping the intervention in between. An exercise diary is recommended.

### 4.2. Future Directions

A randomized controlled study, also including more impaired stroke survivors, is warranted for further investigating the effects of the SIP. It would also be desirable to investigate how the best possible transition from close personal care by therapists in a rehabilitation facility to active continuation of the exercise program, primarily at home, can be achieved using a blended-care model based on the SIP. The study design should address the above-mentioned limitations, such as revised assessment methods with further focus on real-time therapy intensity and frequency, economic analyses and implementation of a validated tool such as the System Usability Scale.

## 5. Conclusions

The SIP is a feasible application-based intervention for less impaired individuals with stroke. It addresses major risk factors, such as a low level of physical activity and hypertension, which are crucial factors in secondary stroke prevention.

## Figures and Tables

**Table 2:** Description of study objectives and outcome measures.

Objectives	Endpoints	Measures and Approaches
Feasibility of the Strokecoach Intervention Program (study methods)	Recruitment	- Number of eligible individuals with stroke enrolled in study - Evaluation of successful recruitment
	Retention and dropout	- Baseline measurement rate was defined as the proportion of eligible patients, who provided informed consent and completed at least one questionnaire - Retention was defined as the proportion of participants, who completed the SIP - Attrition from intervention: Proportion of enrolled participants, who prematurely terminated their participation
To explore the acceptability of using the Strokecoach intervention consisting of Training, Coaching and Monitoring	User engagement	- Mean intervention days of patients
	Acceptability and satisfaction of coaching aspects (webinars, personal coach)	- Quality ratings of personal coach - Satisfaction of participants with webinars: ▪ attendance: proportion of participants, who participated at least one webinar ▪ rating of webinars (1 to 10, higher scores representing more importance) and their duration (3-point Likert scale)
	Acceptability of monitoring	- Planned/actual measures of blood pressure and physical activity measures
To provide preliminary evidence for patient-reported outcome measures and performance-based measures in a home-based setting.	Health-related quality of life	- German version of Short Form-36 (SF-36) [39]
	General health	- Questions related to health (walking, speaking, vision, memory, concentration, mood, sleep, current health) on a 10-point rating scale
	Functional mobility	- Timed Up and Go [40]
	Walking speed	- 4 m walking test [41]
	Muscle function/strength	- Self-performed rating of muscle strength evaluation for six major muscle groups
To explore physiological parameters and physical activity patterns	Blood pressure	- Mean blood pressure per day - Mean alerts during the intervention period
	Daily physical activity during intervention period	- Weekly average of steps per day during intervention period

SIP: Strokecoach Intervention Program.

**Table 3:** Patient characteristics (*n* = 96).

Sex (% female)	54.2%
Age (in years) Mean (SD)	54.8 (13.1) missing *n* = 44
Type of stroke	ischemic: 52.5% haemorrhagic: 21.3% not known: 13.8% TIA: 3.8% missing *n* = 16
Time since stroke (in years) Mean (SD)	6.1 (6.6) missing *n* = 18
Affected body side (subjectively)	left 50.7% right 40.0% both 5.3% missing *n* = 21
Recurrent stroke prior to participation (% yes)	35.0% missing *n* = 16

**Table 4:** Subgroup baseline characteristics (*n* = 38).

		Total
		*n* = 38
Sex, female (%)		57.9%
Age in years, M (SD) missing *n* = 15		49.9 (11.6)
Stroke subtype (%)	ischemic	57.9%
	haemorrhagic	15.8%
	Not known	15.8%
	TIA	2.6%
	other	7.9%
Time since stroke (in years), M (SD)		6.4 (6.9)
Subjectively affected body side missing *n* = 1		Left: 43.2%, right: 54.1%, no affected side: 2.7%
Recurrent stroke (% yes)		31.6%
mRS score Md [IQR]		2 [1]
intervention duration, in days M ± SD (Range)		92.6 ± 5.1 (61; 141)
function arm [1–10], Md [IQR]		8.5 [4.0]
strength arm [1–10], Md [IQR]		6.0 [4.0]
pain arm [1–10], Md [IQR]		7.0 [3.0]

Legend: Md = median, M = mean, IQR = interquartile range, SD = and standard deviation.

## Data Availability

The datasets used and analyzed during the current study are available from the corresponding author upon reasonable request.

## Supplementary Materials

The following supporting information can be downloaded at: https://www.mdpi.com/article/10.3390/bioengineering11020135/s1, Table S1: Further characteristics; Table S2: Analysis of SF-36 pre- and post-intervention results (*n* = 38); Table S3: General health questions results pre- and post-intervention (*n* = 38); Table S4: Pre- and post-intervention results of the Timed Up and Go test and walking speed.

## Abbreviations

SD	Standard deviation
SIP	Strokecoach Intervention Program
TUG	Timed Up and Go
