# Supplementary material for: Feasibility of an Application-Based Outpatient Rehabilitation Program for Stroke Survivors: Acceptability and Preliminary Results for Patient-Reported Outcomes
Source: Bioengineering (Basel). 2024 Jan 29;11(2):135. doi: 10.3390/bioengineering11020135 (PMC10886035; doi:10.3390/bioengineering11020135)
Supplement: Supplementary file 1 [file bioengineering-11-00135-s001.zip › bioengineering-2807567-supplementary.pdf]

Table S1. Further characteristics.

Characteristic		Frequency (in %)
Number of medication	No medication	13.2%
	1 to 4	44.7%
	≥5	42.1%
Medication Groups	Analgetic	7.9%
	Antihypertensive	55.3%
	Antiplatelets/Anticoagualtes	71.1%
	Statins	42.1%
	Anti-diabetic	7.9%
	Anti-depressants	26.3%
	Anti-inflammatory	7.9%
	Cholesterol-lowering medication	2.6%
	Thyroid medication	5.3%
	Proton pump inhibitors	21.1%
	Anti-rheumatic	2.6%
	Anti-epileptic	5.3%
	Muscle relaxant	15.8%
	Immunsuppressiva	2.6%
	Other	26.3%
Comorbidities	No comorbidities	14.8 %
	Diabetes	18.5 %
	Peripheral vascular	14.8 %
	Cerebrovascular disease	18.5 %
	Congestive heart failure	11.1 %
	Respiratory disease	7.4 %
	Tumor	11.1 %
	Rheumatologic disease	18.5 %
	Musculoskeletal problems	18.5 %
	Renal disease	7.4 %
	Psychiatric problems	11.1 %
	Other	18.5 %
		missing $n = 11$
Affected areas	Gait	78.9%
	Hand and arm function	71.1%
	Concentration and/or memory	57.9%
	Pain	36.8%
	Mood	39.5%
	Speaking	34.2%
	Vision	31.6%
	Other	15.8%

Legend: Self-reported information. Multiple responses were possible.

Table S2. Analysis of SF-36 pre- and post-intervention results (n = 38).

	**pre**	**post**	**Statistics**			
	M ± SD	M ± SD	df	t-Test	Wilcoxon signed-rank	p-value
Bodily Pain	61.8 ± 26.6	68.0 ± 23.2	37	NA	W = 83.5, z = −1.900	0.059
Physical Functioning	51.7 ± 25.9	60.8 ± 26.6	35	NA	W = 91.5, z = −2.725	0.006
Mental Health	59.9 ± 23.6	71.9 ± 18.6	36	t = −4.249	NA	<0.001
Role limitations due to emotional health	57.9 ± 45.0	63.6 ± 46.2	37	NA	W = 91.0, z = −0.523	0.607
Vitality	44.4 ± 21.9	57.0 ± 20.5	37	t = −5.572	NA	<0.001
Social functioning	66.7 ± 31.1	73.6 ± 26.7	35	NA	W = 93.0, z = −1.088	0.265
General health	52.8 ± 15.4	53.1 ± 18.4	37	NA	W = 270.5, z = −0.729	0.468
Role limitations due to physical health	37.5 ± 35.7	45.6 ± 42.6	37	−1.107	NA	0.275

Legend: Bold are statistically significant results; df = degrees of freedom, t-test or Wilcoxon signed-rank test.

Table S3. General health questions results pre- and post-intervention (n = 38).

	**Pre**	**post**	**Statistics (Wilcoxon Signed Rank-Test)**			
**[0–10]**	**Md [IQR]**	Md [IQR]	df	W	z	p
Walking	6 [3]	7 [4]	36	89.0	−2.197	0.027
Speaking	9 [1]	9 [3]	36	107.0	−0.295	0.769
Mood	8 [4]	8 [3]	36	104.5	−1.803	0.066
Vision	8 [3]	8 [3]	36	151.0	−0.913	0.358
concentration	7 [4.25]	8 [3]	35	90.5	−1.937	0.048
Sleep	8 [4]	8 [2]	36	193.0	−1.078	0.267

Current health	6 [3[	7 [2]	36	141.0	−1.882	0.052
memory	7 [4]	8 [4]	35	142.0	−0.851	0.394

Legend: df = degrees of freedom; Md = Median; IQR = interquartile range.

Table 4. Pre- and post-intervention results of the Timed Up and Go test and walking speed.

		**Total (*n* = 38)**	**MRS 1 (*n* = 11)**	**MRS 2 (*n* = 23)**	**MRS 3, 4 (*n* = 4)**
		M ± SD	M ± SD	M ± SD	M ± SD
Timed Up and Go (in s)	pre	13.3 ± 4.9	11.4 ± 4.9	13.0 ± 3.8	20.1 ± 5.7
	post	14.2 ± 9.9	9.4 ± 2.3	13.6 ± 5.5	30.8 ± 22.5
	Statistics (Wilcoxon signed-rank test)	W = 303.5, p = 0.857	W = 35, p = 0.476	W = 107, p = 0.781	NA
Gait speed (in m/s)	pre	0.67 ± 0.27	0.86 ± 0.32	0.65 ± 0.19	0.36 ± 0.11
	post	0.69 ± 0.40	0.94 ± 0.60	0.60 ± 0.24	0.49 ± 0.30
	Statistics (t-test)	t = -0.310, df = 31, p = 0.759	T = -0.63, df = 8, p = 0.546	t = 1.060, df = 18, p = 0.303	t = -1.326, df = 3, p = 0.277

Legend: Gait speed was measured with the 4m gait test. M = mean, SD = standard deviation, df = degrees of freedom.
